# Metastatic Adenocarcinoma of the Lung Mimicking Miliary Tuberculosis and Pott’s Disease

**DOI:** 10.7759/cureus.12869

**Published:** 2021-01-22

**Authors:** Dawlat Khan, Muhammad Umar Saddique, Theresa Paul, Khaled Murshed, Muhammad Zahid

**Affiliations:** 1 Internal Medicine, Hamad Medical Corporation, Doha, QAT; 2 Internal Medicine, Hamad General Hospital, Doha, QAT; 3 Pathology, Hamad General Hospital, Doha, QAT; 4 Medicine, Hamad Medical Corporation, Doha, QAT

**Keywords:** potts disease- tuberculous spondylitis, spinal mass lesion, miliary pattern, adenocarcinoma lung

## Abstract

Tuberculous spondylitis (Pott’s disease) is among the frequent extra-pulmonary presentations of tuberculosis (TB). The global incidence of lung adenocarcinoma is on the rise, and it is a rare differential diagnosis of miliary shadows on chest imaging. It has a predilection to metastasize to ribs and spine in particular. There is a very close clinical and radiological resemblance in the presentation of spinal metastasis of lung cancer and Potts’s disease. It poses a diagnostic challenge to clinicians particularly in TB endemic areas to arrive at an accurate diagnosis, leading to disease progression and poor outcome. We report a 54-year-old female patient presented with constitutional symptoms of on and off fever and back pain. Her chest X-ray revealed miliary shadows, and acid-fast bacilli (AFB) sputum smear and TB polymerase chain reaction (PCR) test came negative; radiological diagnosis of tuberculous spondylitis was done on computerized tomography (CT) chest and magnetic resonance imaging (MRI) spine. Subsequent bronchoscopy and bronchoalveolar lavage (BAL) cytology showed malignant cells and CT-guided lung biopsy confirmed lung adenocarcinoma with spinal and brain metastasis. Despite being started on chemo-immunotherapy and radiotherapy her outcome was poor due to advanced metastatic disease. This case highlights the significance of considering metastatic adenocarcinoma of the lung a rare but ominous possibility in the differential diagnosis of miliary shadows on chest imaging. Early bronchoscopy and biopsy must be considered in all patients presenting with miliary pulmonary lesions and spinal lesions to make a correct diagnosis, preventing an unnecessary delay in starting proper treatment and poor outcome. It also emphasizes the importance of better understanding the different radiographic features of the two common mimics, spinal tuberculosis, and metastatic spinal tumors.

## Introduction

Tuberculosis is a granulomatous disease caused by Mycobacterium tuberculosis (M. tuberculosis). Although the major disease burden rests in developing countries, the human immune deficiency virus (HIV) pandemic has led to a high global TB prevalence [1]. Skeletal TB accounts for 10-35% of cases of extra-pulmonary TB globally, and the most common form of skeletal TB is tuberculous spondylitis - Pott’s disease [2]. Back pain, tenderness, neurological deficits depending upon the site of spinal involvement, and constitutional symptoms, e.g., fever, fatigue, and weight loss, are common clinical manifestations [3]. Pott’s disease’s considerable differential diagnosis is degenerative disc disease, osteopenic vertebral body collapse, pyogenic spinal infection, and primary or secondary malignancies [4]. Amongst the malignancies, lung adenocarcinoma is the most prevalent cancer that metastasizes to the spine. It inclines to involve the thoracic and lumbar spine and may mimic Pott’s disease [5,6]. Adenocarcinoma of the lung, among others, is a potential differential diagnosis of miliary shadows on chest imaging [7]. Because of close radiological resemblance, it is often a challenge to differentiate Pott’s disease from primary or early metastatic spinal tumors. It is a common cause of misdiagnosis and may result in serious and irreversible neurological sequelae and overall poor patient outcome [8].

## Case presentation

A 54-year-old female presented with a two-month history of left scapular and thoracic back pain, which started after slipping on the floor and hitting her back. She reported constant, dull pain of 4/10 severity, aggravated with movements and deep inspiration, partially relieved with analgesics that affected her daily life activities. There was no associated limb weakness, numbness, or problems with bowel and bladder control. She denied having fever, night sweats, weight loss, cough, shortness of breath, headache, nausea, or vomiting. There was no personal or family history of TB or any chronic medical illness. She was a non-smoker and had no history of high-risk sexual contacts or intravenous drug abuse.

Clinically she was oriented, alert, and hemodynamically stable. Physical examination was remarkable for tenderness on palpation of the T3-T12 vertebrae and left scapula. Power both proximal and distal, and reflexes were normal in all extremities. Bilateral downgoing plantar responses were noted. The sensory examination was normal in all extremities. The chest was clear on examination. The rest of the examination was unremarkable.

Investigations were significant for mild leukocytosis and elevated alkaline phosphatase. Results of routine investigations are shown in Table 1. Human immune deficiency virus, Hepatitis B and C serology was negative. TB Quantiferon was also negative.

**Table 1:** Routine blood investigations INR: International normalized ratio; ALT: Alanine transaminase; AST: Aspartate transaminase.

Laboratory parameters	Patients values	Reference range
White cell count	14.7	4-111 x 10^3 ^/µl
Hemoglobin	14.9	12-15 gm/dl
Platelets	352	150-450 x 10^3 ^/µl
INR	0.9	1.0
Creatinine	40	53-97 µmol/L
Urea	8.10	2.76-8.07 mmol/L
Sodium	136	135-145 mmol/L
Potassium	4.8	3.6-5.1 mmol/L
Bicarbonate	26	24-30 mmol/L
Calcium	2.30	2.10-2.60 mmol/L
Total Bilirubin	18	0-21 µmol/L
Total protein	64	66-87 gm/L
Albumin		35-52 gm/L
ALT	9	0-30 U/L
AST	10	0-31 U/L
Alkaline phosphatase	231	35-104 U/L

**Figure 1:**
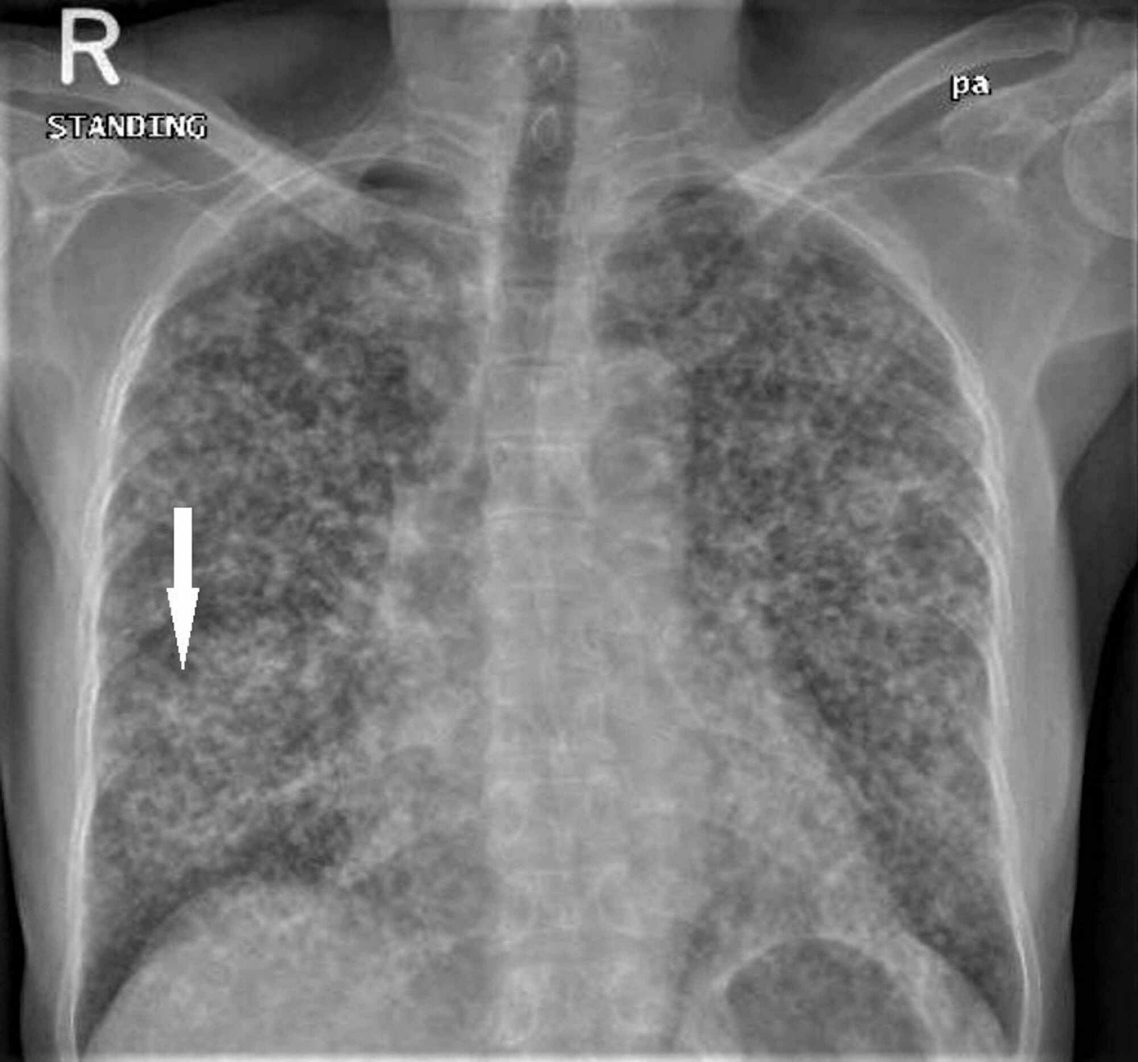
Chest X-Ray (CXR) Posteroanterior (PA) chest X-ray view depicting bilateral miliary shadows and right lung suspicious infiltrates (Arrow).

Chest X-ray (CXR) revealed faint, reticulonodular infiltrates distributed uniformly throughout the lung fields bilaterally (Figure 1). The initial clinical impression was of miliary pulmonary TB based on CXR findings and coming from a TB endemic area. She was kept in a negative pressure room, and sputum was sent for acid-fast bacilli (AFB) and TB polymerase chain reaction (PCR); both reported negative.

**Figure 2:**
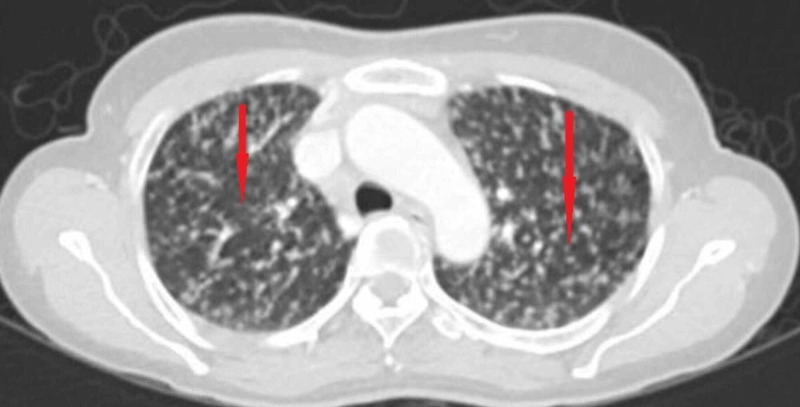
Chest CT scan Chest computerized tomography (CT) scan showing bilateral numerous miliary nodules, some are indicated by red arrows.

Subsequent CT-chest confirmed bilateral miliary nodular lung infiltrates and an osteolytic lesion at the T-2 vertebral body, encroaching on the spinal canal (Figure 2).

**Figure 3:**
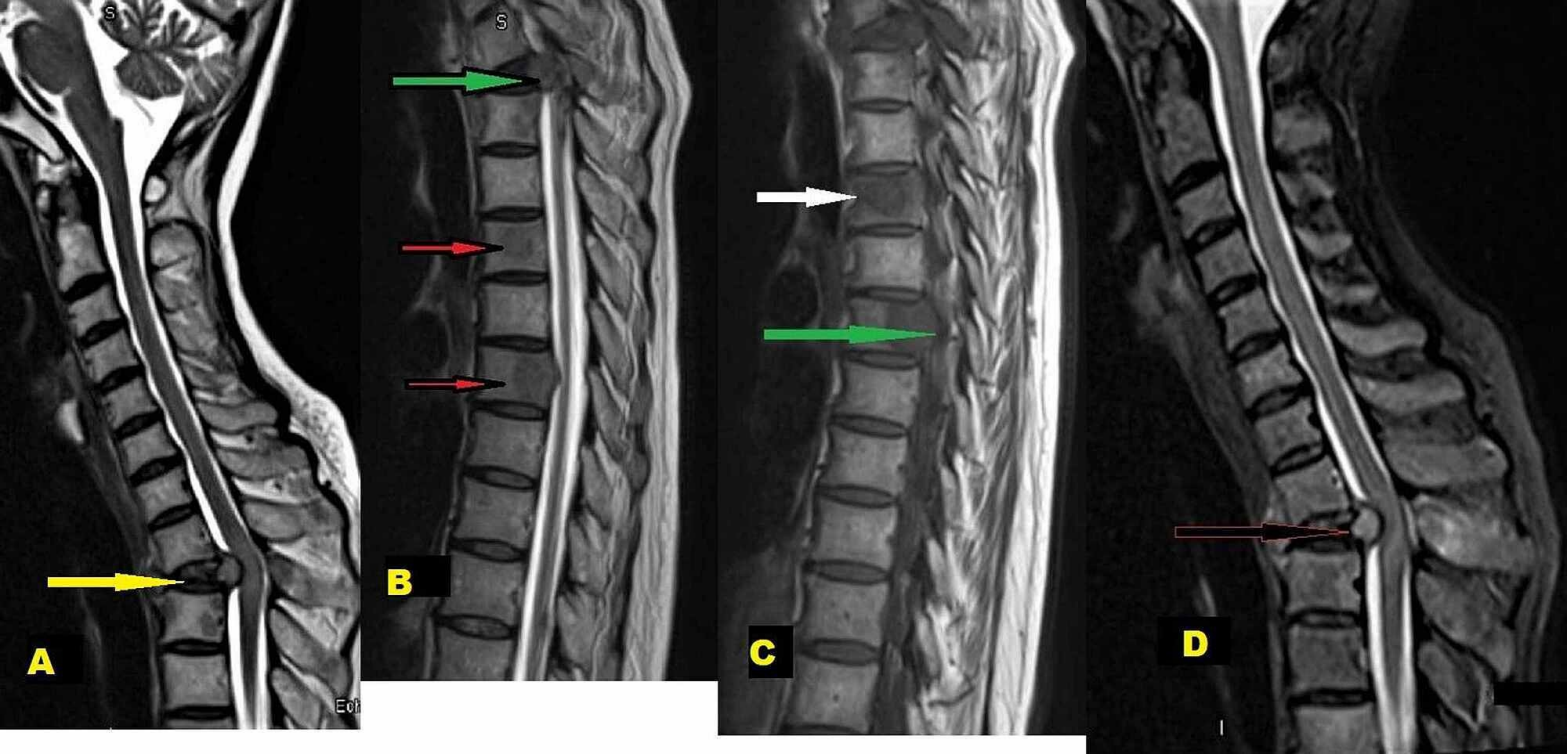
MRI Spine A) (Para-sagittal view) T2-weighted images showing compression at T1-T2 spinal level (yellow arrow) B) Multiple spinal bony metastasis (red arrows) with compression on the anterior spinal cord (green arrow) C) T1-weighted MRI images showing body metastatic (white arrow) and pedicles metastatic involvement (green arrow) D) Extradural compression on the cord pushed posteriorly (arrow)

An urgent contrast-enhanced magnetic resonance imaging (MRI) of the whole spine showed severe T2 vertebra signal changes and post-contrast enhancement, structural collapse, compression fractures, and significant compression of spinal cord opposite T2 level, and pronounced focal osseous lesions at T7 (Figure 3A-3D).

Based on CT chest and MRI spine findings, the primary radiological diagnosis was miliary pulmonary TB with extra-pulmonary TB involving spine - Pott’s disease. MRI spine findings were primarily considered as tuberculous spondylitis with atypical, more central vertebral body involvement patterns.

**Figure 4:**
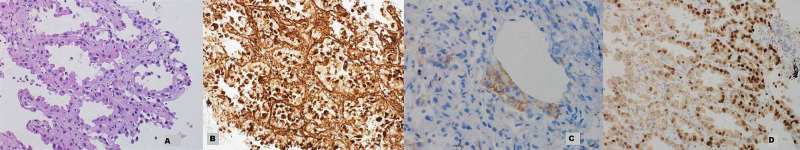
Histopathology slides A) Photograph depicting tumor arranged in glands lined by atypical cells (Hematoxylin & Eosin stain, x200) B) Tumor cells are immunoreactive for Napsin-A C) PDL-1 shows weak to moderate membranous staining in the tumor cells D) Tumor cells demonstrate nuclear positivity for TTF-1

Two sputum samples for AFB and TB PCR were reported negative. To confirm these miliary nodules’ nature, a bronchoscopy was arranged that depicted normal bronchial mucosa and no endobronchial lesion. Bronchoalveolar lavage (BAL) was negative for TB-workup, but cytology revealed malignant cells. The histopathology of CT-guided lung biopsy confirmed malignant epithelial cells within lymphatics consistent with lymphatic carcinomatosis. The immunohistochemical profile was positive for thyroid transcription factor-1 (TTF-1), napsin A, CK7, Ber-Ep4, Calretinin, and negative for anaplastic lymphoma kinase (ALK) (5A4). Programmed death-ligand 1 (PD-L1) was weakly positive (1+), and epidermal growth factor receptor (EGFR) L858R was detected in exon 21 (Figure 4). These findings confirmed metastatic primary adenocarcinoma of the lung.

**Figure 5:**
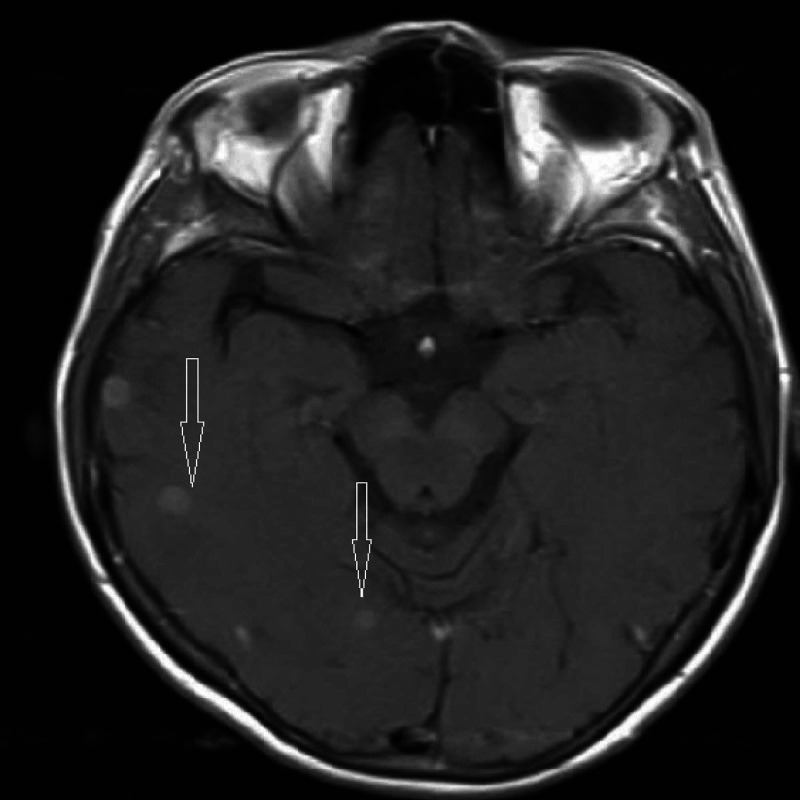
MRI-Brain MRI-brain showing brain metastasis (arrows)

MRI brain revealed innumerable small metastasis in both cerebral hemispheres, cerebellar hemispheres, and the brainstem (Figure 5).

Upon the oncology multidisciplinary team’s decision, she was started on palliative chemo-immunotherapy and whole-brain radiotherapy for brain metastasis. No active neurosurgical intervention was done due to advance spinal metastatic disease, and she was treated with spinal radiotherapy. She received an epidermal growth factor receptor (EGFR) inhibitors-based immunotherapy.

Initially, she had slight improvement, but she developed paraparesis and bowel incontinence after two months of treatment and needed assistance to ambulate. Despite being on active immunotherapy and spinal rehabilitation, there is no significant improvement in functional status.

## Discussion

Tuberculosis (TB), along with HIV and malaria, is a dangerous disease combination globally. Mycobacterium primarily infects the lungs, but secondary to hematogenous spread, other organs are involved as extra-pulmonary TB (EPTB) [8,9]. Globally, skeletal TB amounts to 10-35% of EPTB cases, and half of the skeletal TB cases are tuberculous spondylitis - Pott’s disease [2]. Spinal TB is a very ancient human disease; Sir Percivall Pott described it in 1779 (Pott’s disease) [10]. Pott’s disease has a predilection for the lower thoracic and lumbar spine. Patients classically present with progressive back pain that lasts for several months, tenderness, neurological deficits, and constitutional symptoms of fever, night sweats, fatigue, and weight loss [3].

Miliary TB forms millet size (1-2 mm) granulomas in various body organs resulting from the massive lympho-hematogenous spread. Miliary TB accounts for less than 2% of all TB cases in immune-competent adults and 20% of EPTB cases [11]. Risk factors for miliary TB are immune-compromised state, diabetes, smoking, alcohol dependence, pregnancy, and underlying malignancy. It is associated with high mortality, even though effective therapy is available [12].

Numerous fine granular or small nodular (1-3 mm) opacities are characteristic findings on chest images of pulmonary miliary TB. Sarcoidosis, histoplasmosis, pneumoconiosis, and rarely metastatic primary lung cancer are the main differential diagnoses of miliary infiltrate on chest radiologic images. High-resolution CT-chest is often useful to narrow down the differential diagnosis. The centrilobular and perilymphatic pattern indicates infectious bronchiolitis and sarcoidosis, respectively, while the random pattern is seen in hematogenous cancer metastasis [7]. Our patient had numerous small nodular bilateral pulmonary infiltrates on CXR and a random nodular distribution pattern on the CT chest.

Lung cancer is the most commonly diagnosed cancer and the world’s major cause of cancer-related deaths. Smoking is a well-established risk factor. Lung cancers are classified into small cell lung cancer (SCLN) and non-small cell lung cancer (NSCLN). Adenocarcinoma is the most prevalent subtype of NSCLC in non-smokers and women [13]. The common presentation is peripherally located solid nodules/mass with pleural involvement but rarely presents a miliary pattern. The close clinical and radiological presentation of miliary pulmonary TB and adenocarcinoma lung presenting as miliary shadows on chest images poses a diagnostic challenge. Without a tissue biopsy, it may lead to misdiagnosis, serious sequelae, and poor outcomes due to completely different treatment modalities [7].

The invention of targeted treatment modalities has led to increased cancer patients' survival, but the metastatic disease still carries a poor prognosis. Bone is the most common site of distant metastasis and is a negative prognostic factor [14]. In a recent retrospective study of 2021 lung cancer patients, one-fourth of patients had bone metastasis, and adenocarcinoma (62.1%) was the most prevalent pathological subtype metastasized to bones. Ribs, thoracic and lumbar spines are the most frequent sites of metastasis. It is attributed to the extensive venous anastomosis and proximity of these organs. High levels of CA-125 and alkaline phosphatase (ALP) were the possible predictors of multiple bone metastasis [5].

MRI spine is the radiological investigation of choice when TB spondylitis or metastasis is suspected. Neoplastic infiltration usually presents as a destructive bone lesion with a well-preserved disc space and sharp endplates or the involvement of only one vertebral body. A destructive bone lesion, along with a poorly defined vertebral body endplate, with or without loss of disc height, indicates an infective process [15]. In the early stages, these changes are less conspicuous and not easily detectable. Our patient's radiological findings were not clear, and the most likely radiological diagnosis was TB spondylitis. Sophisticated image modalities are used to differentiate between these mimics [16].

There is a case in literature where spinal metastasis from lung cancer in TB endemic areas was mistaken for Pott's disease. These patients were treated with anti-tuberculous medications, neuro-surgical interventions, and led to a delay in the diagnosis of lung cancer and unfavorable clinical outcome [16]. On the contrary, in low TB prevalence countries, spinal tuberculosis was mistakenly diagnosed as suspected metastatic cancer and treated with radiotherapy. Delay in diagnosing TB in such cases led to disseminated TB, which carried a poor prognosis [8].

Our patient belonged to a TB endemic area, had miliary shadows on the chest imaging, and a preliminary radiological diagnosis was TB spondylitis. The sputum examination was negative for AFB twice in miliary TB. In resource-limited countries where bronchoscopy is not readily available, and the diagnosis of TB relies mainly on imaging and clinical suspicion, then it might lead to the wrong treatment. But on-time bronchoscopy and cytology examination of the bronchoalveolar lavage clenched the correct diagnosis, which was later confirmed on lung biopsy. A multidisciplinary team meeting was held, and a comprehensive treatment plan was arranged, which was mainly supportive care.

Key learning points

Metastatic adenocarcinoma of the lung must be considered as a potential differential diagnosis of miliary shadows on chest images. There should be a low threshold for doing bronchoscopy and biopsy.

The guidelines for patient care of miliary lesions on chest images must be revised and updated for a good clinical outcome.

Early spinal metastases of adenocarcinoma lung and Pott’s disease are common mimics of each other. Sophisticated imaging modalities must be used if conventional imaging studies do not help arrive at an accurate diagnosis.

Premature closure of clinical diagnosis is a common cause of delayed diagnosis. It can be avoided by aggressively pursuing the appropriate differential diagnosis and applying a thorough clinical decision-making approach.

## Conclusions

This case highlights the importance of considering metastatic adenocarcinoma of the lung as a considerable differential diagnosis of the miliary pattern on chest images. Although miliary and spinal TB can occur simultaneously, the metastatic disease should always be considered until a diagnosis is made. There is an inclination in TB endemic areas to treat these patients empirically with antituberculous therapy despite negative sputum smear. This premature closure leads to a delay in accurate diagnosis. In the case of miliary shadows on chest images, every effort must be made to get a bronchoscopy done and a tissue biopsy obtained to confirm the diagnosis. Authors also empathize on the better understanding of the different radiographic features of the two common mimics, spinal tuberculosis and metastatic spinal tumors. The use of sophisticated radiological modalities must be encouraged when the features are less obvious on conventional spinal imaging studies. These efforts will lead to accurate diagnosis, appropriate treatment, and favorable patient outcomes.
